# Too much safety? Safeguards and equal access in the context of voluntary assisted dying legislation

**DOI:** 10.1186/s12910-020-00483-5

**Published:** 2020-05-13

**Authors:** Rosalind McDougall, Bridget Pratt

**Affiliations:** Centre for Health Equity, Melbourne School of Population and Global Health, University of Melbourne, Level 4, 207 Bouverie St, Melbourne, VIC 3010 Australia

**Keywords:** Voluntary assisted dying, Medical assistance in dying, Equal access, Equity

## Abstract

**Background:**

In June 2019, the Australian state of Victoria joined the growing number of jurisdictions around the world to have legalised some form of voluntary assisted dying. A discourse of safety was prominent during the implementation of the Victorian legislation.

**Main text:**

In this paper, we analyse the ethical relationship between legislative “safeguards” and equal access. Drawing primarily on Ruger’s model of equal access to health care services, we analyse the Victorian approach to voluntary assisted dying in terms of four dimensions: horizontal equity, patient agency, high quality care, and supportive social norms. We argue that some provisions framed as safeguards in the legislation create significant barriers to equal access for eligible patients.

**Conclusions:**

While safety is undoubtedly ethically important, we caution against an overemphasis on safeguarding in voluntary assisted dying legislation given the implications for equal access.

## Background

Developing and implementing a system for voluntary assisted dying is a highly complex challenge currently facing the health sector in Victoria, Australia. There is a growing momentum internationally around voluntary assisted dying (VAD) [1]. Some form of voluntary assisted dying is permitted in the Netherlands, Canada, Belgium, Colombia, Switzerland and Luxembourg as well as in ten states of the US, and Victoria recently joined this group of jurisdictions [2, 3]. In November 2017, the Victorian parliament passed legislation to permit competent terminally ill patients to self-administer a lethal medication. A doctor can administer the medication only if the patient is physically incapable of taking the medication themselves. The *Voluntary Assisted Dying Act 2017 (Victoria)* came into effect on 19 June 2019, and its implementation is being closely followed by policymakers in other Australian states where similar reforms are being considered [4], and in the state of Western Australia where similar legislation was passed in December 2019. Elsewhere in the world, medical assistance in dying is a topic of current debate and the focus of attempts at legislative change (e.g. [5, 6]).

A discourse of safety is prominent in relation to the Victorian legislation. The message that the legislation is the “safest and most conservative in the world” is repeatedly used by government, including throughout the legislative debate and implementation period [7, 8]. Using the concept of “safeguards” is common in these discussions; the claim that the legislation contains “68 safeguards” is consistently made by government and commentators [7, 9–12]. With the very recent debate in the state of Western Australia, there is evidence that safeguarding is becoming competitive: the Western Australian government presented their voluntary assisted dying legislation as containing 102 safeguards [13].

At the same time, stories of eligible Victorian patients struggling to access voluntary assisted dying are emerging in the media [14–16]. In these stories, the limited number of doctors willing to provide voluntary assisted dying is reported as a key barrier, alongside the extensive administrative requirements of the legislated process.

In this paper we analyse the ethical relationship between equal access and legislative provisions aimed at safety, in the voluntary assisted dying context. Our focus is on equal access to VAD for *eligible* patients. In the Victorian legislation [17], the eligibility criteria capture only competent local adults with terminal illness. In order to be eligible, a patient must be a Victorian resident aged 18 or over, with decision making capacity in relation to voluntary assisted dying. They must have an incurable condition expected to cause death within 6 months (12 months for neurodegenerative conditions), that is “causing suffering to the person that cannot be relieved in a manner that the person considers to be tolerable”. ([17],p.15) The Victorian regime thus excludes some types of patients that are included in the scope of voluntary assisted dying in other jurisdictions, such as patients who are not terminally ill or those requesting via advance directive. These patients’ potential entitlement, while beyond the scope of this paper, is important to consider and there has been extensive and continuing ethical discussion about who ought to be eligible for voluntary assisted dying (e.g. [18–22]). However, our focus in this paper is not on equal access in the sense of the justifiability of the Victorian eligibility criteria. Rather, we analyse whether the Victorian legislation promotes equal access to voluntary assisted dying for the patient group that legislators have identified as entitled to request this intervention.

We argue that some provisions framed as “safeguards” in the legislation have substantial consequences for equal access. We begin by outlining the requirements of the *Voluntary Assisted Dying Act 2017 (Victoria)*, and the ways in which the concepts of safety and access have been utilised during the development of the Victorian approach*.* Then, drawing primarily on Ruger’s model of equal access to health services [23–25], we analyse the Victorian legislation in terms of four dimensions of equal access: horizontal equity, patient agency, high quality of care, and supportive social norms. Our view is that legislation about healthcare – as one element of an overall health system – ought to be concerned with equal access. We argue that various safeguards in the Victorian approach create significant barriers to equal access. While safety is undoubtedly ethically important, our analysis indicates that a legislative focus on maximizing safety comes at the expense of equal access.

Given our use of Ruger’s model in this context, it is important to clarify terminology. Ruger uses the term “health care services” to refer to health care. However, in discussions of VAD, the term “health services” is often used in the sense of the hospitals and other organisations that deliver health care. For example, in the glossary of the VAD guidance document for health practitioners, health service is defined in the sense of organisations: “includes hospitals, community health services, primary care health services, residential aged care services and other organisations that provide health care” ([26], p.vii). In this paper, when we use the term health care services in relation to VAD, we mean the provision of VAD-related care through the health system; we use “health care services” in the sense of care rather than the in the sense of organisations. We have chosen to use the term in line with Ruger’s usage, given the role of her model as the conceptual scaffold for our analysis.

### The Victorian legislation for voluntary assisted dying

Understanding the key features of the Victorian legislation is the first step in analysing the relationship between legislative safeguards and equal access. The main elements of the *Voluntary Assisted Dying Act 2017 (Victoria)* are summarised in Table 1. In Victoria, the legislated process for requesting voluntary assisted dying requires at least three formal requests to be made by the patient. Two senior doctors are involved. The lead doctor is the “co-ordinating practitioner” who assesses the patient’s eligibility and prescribes the medication (or administers the medication if the patient is incapable of taking the medication). The “consulting practitioner” conducts a second assessment of the patient’s eligibility. Approval by the state’s Department of Health and Human Services is also necessary.

**Table 1:** Key features of the Voluntary Assisted Dying Act 2017 (Victoria) [27]

To access VAD, a patient must meet all of the eligibility criteria.	• be an adult Australian citizen or permanent resident and reside in Victoria, • have decision-making capacity in relation to voluntary assisted dying, • have a medical condition that is incurable, advanced, and progressive, • have a prognosis of no longer than 6 months (or 12 months in the case of neurodegenerative disease), and • be experiencing suffering caused by the relevant condition, that cannot be relieved in a manner that the patient deems tolerable.
The patient needs to make at least three formal requests for VAD.	A minimum of two verbal requests and one written request
For eligible patients, the medical practitioner will write a prescription for a lethal medication that the patient can ingest at a time of their choosing.	Only for patients who are unable to self-administer, the VAD medication can be administered by the physician.
The patient must be assessed by two medical practitioners.	• The medical practitioners must be either a vocationally registered general practitioner (GP) or a specialist, and have completed the approved VAD assessment training. • At least one must have held their specialist fellowship or be a vocationally registered GP for a minimum of 5 years. • At least one must have expertise in the relevant condition.
Health practitioners can conscientiously object to participating in any or all of the processes involved in providing voluntary assisted dying.	
Health practitioners must not initiate discussion about VAD with a patient, and must report colleagues whom they reasonably believe have initiated such discussions.	

There is strong protection in the legislation for health practitioners with a conscientious objection to voluntary assisted dying. Section 7 of the Act states that

“[A] registered health practitioner who has a conscientious objection to voluntary assisted dying has the right to refuse to do any of the following – (a) to provide information about voluntary assisted dying; (b) to participate in the request and assessment process; (c) to apply for a voluntary assisted dying permit; (d) to supply, prescribe or administer a voluntary assisted dying substance; (e) to be present at the time of administration of a voluntary assisted dying substance; (f) to dispense a prescription for a voluntary assisted dying substance” ([17], pp.13-14).

There is no requirement for health professionals with a conscientious objection to voluntary assisted dying to refer patients on to a willing practitioner. While health practitioners are not required to provide information about VAD to a patient who requests it, they are instructed in the health practitioner guidance document that “it is important that medical practitioners who choose to refuse to provide information do not impede or obstruct access to voluntary assisted dying” ([26], p.7).

It is worth noting two features of the Victorian legislation that are unique to this jurisdiction, compared with other places internationally that allow voluntary assisted dying [27, 28]. Firstly, the Act requires that discussion of voluntary assisted dying must be initiated by the patient. It is prohibited for health professionals to raise voluntary assisted dying with their patients. Section 8 of the Act states that

“[a] registered health practitioner who provides health services or professional care services to a person must not, in the course of providing those services to the person – (a) initiate discussion with that person that is in substance about voluntary assisted dying; or (b) in substance, suggest voluntary assisted dying to that person” ([17], p.14)

Contravention of this prohibition is “to be regarded as unprofessional conduct” ([17], p.14). The guidance document for health professionals specifies that “[t]he intention of this provision is to protect those who may be open to suggestion or coercion” ([26], p.6).

Secondly, the legislation requires that at least one of the two doctors has expertise and experience in the patient’s condition. Section 10 states that “[e]ither the co-ordinating medical practitioner or each consulting medical practitioner must have relevant expertise and experience in the disease, illness or medical condition expected to cause the death of the person being assessed” ([17], p.17). In the guidance document for health practitioners, this provision is interpreted as “the medical practitioner is required to be a medical specialist in the patient’s medical condition” ([26], p.4).

### Legislative features are framed as safeguards

These features of the legislation, and many others, are presented as “safeguards”. The legislation is consistently described as involving “68 safeguards” (e.g .[7, 9]). This 68 safeguards claim is based on the final report of the Ministerial Advisory Panel [29], which formed the basis of the voluntary assisted dying bill presented to parliament. The report contains a three page section entitled “Safeguard summary” which lists 68 “legislative safeguards” under various headings such as “Access”, “Request”, “Medication management” and so on ([29], pp.181-3). The report also contains an appendix entitled “Safeguards and jurisdictional comparison” ([29], pp.217–228). In this appendix, the proposed Victorian legislation is compared to eight other jurisdictions that allow VAD. The comparison table lists 68 features of the proposed Victorian legislation, and indicates with ticks and crosses which features are present in the other VAD jurisdictions. The fact that none of the comparator jurisdictions includes all of the Victorian features is presumably the basis for the government’s claim that the Victorian legislation is “the safest and most conservative in the world” [7, 8].

While the elements of the legislation within the comparison table are all framed as safeguards, they are not all aimed at *patient* safety specifically. Some, such as the conscientious objection provisions, are explicitly labelled by the Ministerial Advisory Panel as “practitioner protections” ([29], p.219). This is in line with Kelsall’s reflection in the Canadian context that “law regarding euthanasia must safeguard both health care workers and patients from possible abuses in its application” ([30], p.E181). The word “safeguards” is thus used broadly in the Victorian context, to include patient-focused and practitioner-focused elements of the legislation. Diverse provisions of the Victorian legislation – such as the prohibition on health practitioners raising VAD, the requirement for one of the doctors to have expertise in the relevant condition, the strong protection for conscientious objectors, and many others – are all framed as safeguards.

### Equal access to VAD as a goal and expectation

Equal access was an aim of the Ministerial Advisory Panel in developing a suggested legislative regime for voluntary assisted dying. While their final report primarily frames their task of designing VAD legislation as “a balance between *promoting autonomy* and providing appropriate safeguards to protect vulnerable people from abuse” ([29], p.210, italics added), they also highlight the need to balance safety and access: “[t]he Panel has also sought to balance the use of appropriate safeguards by ensuring voluntary assisted dying is practically accessible to Victorians who meet the eligibility criteria” ([29], p.211). This aligns with the valuing of equal access within the Australian health system more broadly. The Australian Charter of Healthcare Rights, for example, states that everyone has the right to be able to access healthcare; patients are told that they should expect to “access services to address [their] healthcare needs” [31].

The published guidance for health practitioners and the information document for people considering VAD both generate a community expectation that eligible patients will be able to access VAD. The state has funded VAD care navigators: two centralised roles aimed at enabling access to VAD. The guidance document for health practitioners states that “the primary role of care navigators is to assist patients who need support in obtaining information about or access to voluntary assisted dying” ([26], p.7). The information document for patients repeatedly presents VAD care navigators as enabling access when a patient’s doctor is not participating or referring:

“you can contact a voluntary assisted dying care navigator to find someone who can help you” ([32], p.10),

“you can contact a voluntary assisted dying care navigator who can link you with the right person” ([32], p.15),

“you can contact a voluntary assisted dying care navigator who will link you with a willing doctor or health practitioner” ([32], p.32).

Thus, in Victoria, equal access to VAD for eligible patients is both a goal of policymakers and a community expectation.

## Main text

### Conceptualising equal access to health services

In the bioethics literature globally, equal access to VAD for eligible patients has not been the subject of detailed ethical analysis. The limited existing ethics literature on access to VAD is concerned with access for marginalized populations (e.g. [33, 34]) or focused on the concept of conscientious objection (e.g. [35, 36]).

There is however a rich philosophical literature on access to health care, including diverse conceptualisations of equal access [37]. We will draw primarily on Ruger’s model of equal access to health care services, which directs attention to patients’ agency and the social conditions in which health care is available and delivered ([23], p.134). Ruger argues that there are four components to equal access that need to be fostered by the health system: horizontal equity, patient agency, high quality care, and supportive social norms [23, 24]. (Each of these components are defined in greater detail in the sections that follow.) Ruger argues that ethical responsibility is shared for promoting these four components of equal access. Multiple societal actors engage in a joint enterprise that by collective action succeeds in co-producing the conditions for all those seeking health care services to be able to access them [25].

Compared to other models of equal access, Ruger’s model reflects a deeper understanding of the essential individual *and* social elements underlying individuals’ wellbeing. On this understanding, unequal access can be addressed not only by patient level interventions to improve health agency and by health professional and facility level interventions to improve the quality of services but also by policies to improve the social and physical environment. Using Ruger’s model to analyse the Victorian VAD legislation enables a comprehensive assessment of the extent to which this legislation promotes equal access. In the sections that follow, we will consider the Victorian legislation in relation to each of the four dimensions of equal access that Ruger proposes.

### Horizontal equity

Horizontal equity occurs when a health care intervention is equally accessible by all patients with a need and desire for that care regardless of, for example, ability to pay or ethnic background. In Ruger’s words, horizontal equity “requires equal treatment for individuals with equal needs”. ([23], p.142) In relation to VAD, horizontal equity would occur when VAD was equally accessible by eligible patients living in rural areas, in regional centres, in the outer suburbs and in the inner city. VAD would be equally accessible by people of different ethnic backgrounds; by men and women; by people who have difficulty making ends meet and by people who are quite wealthy, and so on. It would also be equally accessible by patients across the range of illnesses that might render a person eligible. Horizontal equity would require a fair distribution across the state of participating health practitioners and health facilities that provide VAD.

Two of the safeguards in the legislation work against horizontal equity. One is the requirement that at least one of the doctors is a specialist in the patient’s condition. The limited number of Victorian doctors willing to provide VAD combines with this provision to create a situation in which it is difficult for patients to access VAD. There are many doctors who are broadly supportive of the law, but not willing to be co-ordinating or consulting practitioners. For example, in our survey of over 5000 clinicians across seven Victorian hospitals, 50.7% of medical specialists supported the legislation but only 38.2% were willing to be a consulting practitioner and 21.4% were willing to be a co-ordinating practitioner [unpublished data]. These numbers get even lower once combined with the specialist requirement. Evidence suggests that there are only small numbers of willing doctors in the highly impacted specialties such as oncology and neurology [unpublished data]. The specialist requirement means that VAD is not equally accessible to eligible patients across the range of relevant medical conditions.

The specific content of the conscientious objection provision also impedes horizontal equity. The legislation safeguards health practitioners from participating in VAD, and from having to provide information about VAD or refer patients to other health practitioners who would assist. There is an extensive literature in ethics and health law about the justifiability of legal protections for conscientious objectors in medicine (eg [35, 36]). In the Victorian context, while many practitioners who do not wish to participate in VAD are willing to refer [38], this aspect of the law creates a lottery for patients. It generates a system where some patients will receive support when they raise VAD with a health professional, while other equally eligible patients may receive nothing. This is particularly problematic for patients in rural and regional areas where, firstly, there are fewer health professionals and, secondly, local practitioners may be hesitant to be involved in a morally controversial procedure within their community [39, 40]. Other legislation governing morally controversial procedures protects conscientious objectors while better facilitating equal access by requiring referral to a willing practitioner. Section 8 of the *Abortion Law Reform Act 2008 (Victoria)* requires that any practitioner with a conscientious objection to abortion “refer the woman to another health practitioner, in the same profession, who the practitioner knows does not have a conscientious objection to abortion” ([41], p.19). Choosing a different version of protection for conscientious objectors in the VAD context creates a substantial barrier to equal access for patients.

Wording in the health practitioner guidance document works to limit the impact of this very strong protection for conscientious objectors, encouraging practitioners to focus on patient needs when deciding whether to be involved in VAD. It states that “[w]hile it is important that the medical practitioner considers their decision carefully this should be balanced with the needs of the patient and the suffering they are experiencing” ([26], p.19). Similarly, the guidance states that “[h]ealth practitioners are expected to … respect their patient’s beliefs, values and the choices they make about end-of-life care, even if it conflicts with their own values or religious beliefs” ([26], p.8). Further, while not mandated in the legislation, the VAD care navigator roles were created to assist patients who had approached health practitioners who were not willing to provide VAD. However, the extent to which this is an effective mechanism for facilitating equal access is limited given the small number of willing practitioners and the specialist requirement. It is also unclear whether navigators will be effective at facilitating access and whether they will be able to do so equitably. Their work is limited by a federal law prohibiting inciting suicide through a carriage service (i.e. telephone); media reports indicate that this law is being interpreted to mean that VAD discussions cannot be conducted using phone, email or teleconferencing [14, 42, 43]. This law, combined with the navigators’ location in metropolitan Melbourne, limits their effectiveness in promoting horizontal equity. Further, while figures on the number of patients seeking information or access to VAD are not yet publicly available, it is unclear whether these two positions are sufficient to address the needs of all patients.

Thus, some of the legislative provisions aimed at safety create substantial barriers to eligible patients accessing VAD. Rather than access based on need, morally irrelevant features such as the specific condition limiting the patient’s lifespan and the health professional first approached about VAD impact on an eligible patient’s ability to access VAD.

### Patient agency

Patient agency means that patients can achieve the health goals they value and navigate their own care within the system. The concept of patient agency includes a patient’s

- health knowledge,
- health-seeking skills, and
- ability to engage in effective health decision-making.

In a system that promotes patient agency, patients can act to achieve their health goals. Patients eligible for VAD could make informed judgements and act to achieve an assisted death if that is their chosen pathway. In relation to this dimension of equal access to VAD in Victoria, the key question is ‘can an eligible patient navigate the system to make an effective decision about VAD that is in line with their own goals and see that decision actualised?’

Elements of the Act focus on ensuring two related aspects of agency for eligible patients: their health knowledge and effective health decision-making. Some of the safeguards in the Act promote these aspects of equal access. Sections 4 and 15 of the Act require patients to have decision-making capacity in relation to VAD. Further, the legislation emphasises the importance of providing information about options to requesting patients both as a guiding principle of the Act and in specific requirements. Principle 1c of the Act states that individuals have “a right to be supported in making informed decisions about [their] medical treatment, and should be given, in a manner [they] understand, information about medical treatment options”. ([17], p.12) Sections 19 and 28 describe what information medical practitioners must provide to eligible patients requesting VAD (for example diagnosis, prognosis, treatment options, palliative care options, and so on), and demand that they do so in a way appropriate to patients’ circumstances. These legislative requirements are supported by extensive online guidance and information for both health professionals and patients. Patients’ health knowledge is further promoted by the requirement that VAD discussions and requests involve an accredited interpreter or certified speech pathologist where needed by the patient ([26], p.47).

However, the Act does not attend to the other core dimension of health agency: health seeking skills. This encompasses having skills to effectively navigate the health system to access VAD and being confident in one’s ability to use these skills. The legislation’s emphasis on safety means that the legislated process to access VAD is long and intricate. The process entails numerous steps and permissions. At a minimum, assuming all approached practitioners agree to provide VAD care and find a patient eligible (which are big assumptions), the process consists of: a patient making a first VAD request, a medical practitioner accepting that request, a first assessment of patient VAD eligibility, referral for consulting assessment, a second assessment of patient VAD eligibility, the patient making a written declaration with two witnesses, the patient making a final VAD request, review of the final VAD request by the coordinating practitioner, applying for VAD permits, and the patient accessing VAD. Skills to navigate this process would potentially include being able to articulate and raise the VAD option with multiple practitioners, to get to different health facilities to see multiple practitioners, to set and attend several medical appointments, to complete the relevant paperwork, coordinate two witnesses to be present, and to find a second doctor to approach if the first doctor they approach with a VAD request is not willing to provide or refer them. The skills that individuals need to navigate this process, particularly at a time when they are very sick, are not directly promoted by the Act. The state government’s provision of VAD care navigators is an attempt to address this issue, particularly in relation to finding willing doctors.

Beyond the complex process for accessing VAD, another safeguard that has a substantial impact on patient agency is the prohibition on health professionals initiating discussion of VAD with patients. The guidance for health practitioners elaborates on how this section of the Act is to be interpreted in practice, with detailed examples of patient utterances that would and would not constitute patient requests for information or access to VAD. For example, a patient saying “Can you give me all of the options?” or “Isn’t there something you can do to put an end to this?” are not sufficient to enable a health practitioner to provide information on VAD legally ([26], p.14). Rather, health professionals are to “provide information about the patient’s end-of-life care options, excluding voluntary assisted dying” ([26], p.14). A patient’s statement such as “I would like you to assist me to die” or “Can you help me die?” would allow the health professional to discuss VAD ([26], p.14).

This safeguard compromises patients’ health knowledge and ability to engage in effective decision-making. If patients are unaware of the prohibition on doctors raising VAD, they may assume that VAD is not an available option for them or that they are not eligible. As Johnston and Cameron highlight,

“[w]hile the prohibition may achieve its intention of preventing people from accessing voluntary assisted dying as a result of coercion or undue influence by a health practitioner, it is likely that this will be achieved through the exclusion of a cohort of people who may have been interested but were never made aware that this was an option for them” ([28], p.463).

There is qualitative evidence that clinicians are uncomfortable with this provision, seeing it as a challenge to their paradigm of good doctor-patient communication. Some perceive it as “legislating against free open discussions with our patients” [27]. Some doctors see this aspect of the legislation as disadvantaging the vulnerable, particularly patients from non-English speaking backgrounds or those with low literacy or poor access to technology [27]. Willmott and colleagues argue that this aspect of the legislation will decrease the quality of care that doctors are able to provide to some patients: “the legislative prohibition on health professionals initiating conversations about voluntary assisted dying may, in cases where patients seek information about all EOL [end of life] options, lead to less optimal patient outcomes” [44]. Further, some clinicians are concerned that doctors will avoid end of life conversations with patients for fear of being seen to raise VAD [27]. The prohibition on health professionals raising VAD therefore has a negative impact on two dimensions of equal access: patient agency and high quality care.

### High quality care

A dimension of equal access is that all patients have access to care *of high quality*. The ability to access any care is important but the quality of the care available is also relevant to equal access. Equal access is not achieved if one patient has access to excellent care while another patient with equivalent health needs can only access lesser quality care.

One aspect of high quality care to which the Act does attend is staff training. Supporting staff appropriately is critical to provision of high quality care for patients. The Act requires that medical practitioners who provide VAD must complete VAD assessment training. Evidence from other jurisdictions indicates that provision of information and support for other health professionals and staff is also necessary to high quality care [45], and this is achieved in Victoria with specific information available online for different health professional roles including nurses and allied health practitioners [46] and interpreters [47].

However, many questions remain about exactly what high quality care entails in relation to VAD. Section 5 of the Act specifies general principles that could be interpreted as defining high quality VAD care, for example that “every person approaching the end of life should be provided with quality care to minimise the person’s suffering and maximise the person’s quality of life”, that “[i] ndividuals are entitled to genuine choices regarding their treatment and care”, and that “[a] therapeutic relationship between a person and the person’s health practitioner should, wherever possible, be supported and maintained” ([17], p.12). These principles are called “voluntary assisted dying principles” in the guidance for health practitioners ([26], p.3) but remain at a very general level, relevant across a wide range of health care interventions.

Detailed specification of high quality VAD care may not be appropriate in legislation, and better placed in other contexts. However, this specification has not yet been achieved or communicated. The document entitled “Voluntary assisted dying safety and quality guidance for health services” draws on generic quality frameworks and directs organisations to “develop core safety and quality voluntary assisted dying indicators” ([48], p.43). A concrete understanding of what high quality VAD care looks like in practice still requires further specification. The legislation established the Voluntary Assisted Dying Review Board, and specifies in Section 93 that one of the Board’s functions is “to promote continuous improvement in the quality and safety of voluntary assisted dying” ([17], p.75). However, meaningfully assessing whether patients have access to high quality VAD care will not be possible until indicators and criteria beyond compliance with the legislated administrative process have been formulated.

Further, there is no legislative requirement for health care organisations to support provision of VAD at their facilities. It is up to individual health organisations to decide the extent to which they will support provision of VAD-related care. While not specifically named in the safeguards table, this aspect of the approach to VAD in Victoria is essentially a protection for organisations that do not want to implement VAD – a safeguard for organisations. By safeguarding health organisations’ ability to choose to offer VAD services, patient access to high quality VAD services may be compromised. Three “model of care pathways” are provided for organisations:

- Pathway A is a “single service”: an eligible patient who requested VAD could be fully supported within the organisation.
- Pathway B is a “partnership service” where the organisation “may support and facilitate the request and assessment process but will need to establish partnerships with other health services and refer people to other services to access appropriate specialists”.
- Pathway C is an “information and support service” who will “provide information and/or referrals for people who want to request voluntary assisted dying and, where appropriate, continue to provide support to these people” [49].

Pathway C is seen as covering “health services that have chosen not to provide voluntary assisted dying” [49], such as the Catholic hospitals which play a substantial role in healthcare provision in Victoria [50]. The reasons motivating a health service’s choice of pathway B or C may include quality of care; not all procedures can be provided in all health services when specific staff skills or facilities are required. However, the existence of the different pathways potentially creates a tiered system for VAD care which is incompatible with Ruger’s model of equal access [51]. The quality of care provided to patients will be variable depending on the organisation treating them: some will receive pathway A care, while other patients will receive pathway C care.

This is a significant variability in care, with institutional objection to VAD creating a substantial burden for patients. As Sumner has highlighted in the Canadian context,

“patients who qualify for MAID [medical assistance in dying] constitute a particularly vulnerable population … Delays in accessing MAID will often have the effect of prolonging their suffering, while transfers to other facilities will be particularly onerous and distressing for both patient and family. Patients’ access to MAID may therefore be impaired much more effectively by institutional refusal than by conscientious refusal by individual practitioners” ([52], p.972).

In Victoria, Calvary Health Care have indicated that they

“[w]ill not facilitate or participate in assessments undertaken for the purpose of a patient or resident having access to or making use of the interventions allowed under the Voluntary Assisted Dying Act 2017 (Vic), nor will we provide (or facilitate the provision of) a substance for the same purpose” [50].

Given that equal access requires high quality care across a health system, the differential in care experienced by patients in pathway C organisations is not consistent with equal access. While there may be reasons in favour of protecting organisations in this way, such a safeguard is not compatible with equal access for patients.

### Supportive social norms

Social norms also have an impact on equal access to health care services. Patient choice is shaped by their social environment and its stigmas and taboos. The social acceptability of a choice will impact on health professionals’ and patients’ behaviour, and thus supportive social norms around a particular healthcare option are necessary for patients to access that option.

As has been evident in the parliamentary debates about VAD in Victoria and elsewhere, current norms around voluntary assisted dying are highly contentious. Several elements of the Act directly impact on the social norms around VAD, aiming to create a supportive normative environment. One is Principle 1f, which aims to promote open discussion of dying and individual values: “individuals should be encouraged to openly discuss death and dying and an individual’s preferences and values should be encouraged and promoted” ([17], p.12). This principle contributes to a normative environment that supports individual choice in relation to VAD. Similarly, the report of the Ministerial Advisory Panel makes it clear that the choice of the term ‘voluntary assisted dying’ is deliberate and intended to contribute to supportive social norms around this option. In a section exploring different terminologies, the report states that “[t]he Panel is of the view that it is important to appropriately describe voluntary assisted dying to avoid unnecessary stigmatisation and to ensure the emphasis is on the person” ([29], p.7).

However, some of the safeguards in the legislation have a negative impact on the social acceptability of VAD. The prohibition on health practitioners raising VAD could be seen as creating a stigma around this option, particularly given that no such legislative prohibition exists in Victoria for any other legally available medical option. Further, specification that conscientious objectors need not refer embeds the contentious nature of VAD into the normative environment. These safeguards contribute to stigma around VAD. If VAD is taboo within a setting, interested eligible patients may not feel confident to discuss VAD. An environment that does not stigmatise VAD is necessary to enable equal access.

## Conclusion

While the Act contains some elements that promote equal access, various provisions framed as safeguards create significant barriers to equal access for eligible patients. Three safeguards in particular are in tension with equal access: that conscientious objectors are protected from referring patients on, the prohibition on health practitioners raising VAD, and the requirement that one of the doctors have expertise in the patient’s specific condition. The option for organisations to choose Pathway C also creates a substantial barrier to equal access. Under the Victorian legislation, access is likely to be unequal and to the disadvantage of patients who are being cared for in Pathway C organisations, patients whose doctors have a conscientious objection to VAD, patients whose condition lacks sufficient participating doctors, and patients who are not knowledgeable about VAD as an option.

Overall, the Act emphasises safety at the expense of equal access. For other jurisdictions developing approaches to voluntary assisted dying, we caution against engaging in a discourse dominated by safety. While safety is of course an important value, safeguards have access consequences. Aiming to maximize safety has negative implications for equal access. In our view, a better balance between safety and access is needed in the context of the Victorian legislation.

Developing voluntary assisted dying legislation requires attention to all four aspects of equal access: horizontal equity, patient agency, high quality care, and a normative environment that does not stigmatise this option. In our view, other jurisdictions developing VAD legislation should firstly consider avoiding the legislative features of the Victorian Act which we have identified as in tension with equal access, in order to reduce likely inequalities in access generated by these features. Secondly, from an ethical perspective, engaging in a discourse of maximizing safety is unhelpful in creating an approach to VAD that promotes equal access for all eligible patients.

## Data Availability

Not applicable.

## Abbreviations

VAD: Voluntary assisted dying
GP: General practitioner
